# Interface Characterization of Bimetallic Ti-6Al-4V/Ti2AlNb Structures Prepared by Selective Laser Melting

**DOI:** 10.3390/ma15238528

**Published:** 2022-11-30

**Authors:** Igor Polozov, Anna Gracheva, Anatoly Popovich

**Affiliations:** Institute of Machinery, Materials, and Transport, Peter the Great St. Petersburg Polytechnic University (SPbPU), Polytechnicheskaya, 29, 195251 St. Petersburg, Russia

**Keywords:** additive manufacturing, powder bed fusion, multimaterial printing, titanium alloys, bimetallic structure

## Abstract

Additive Manufacturing (AM) of multimaterial components is a promising way of fabricating parts with improved functional properties. It allows for the combination of materials with different properties into a single component. The Ti_2_AlNb-based intermetallic alloy provides high temperature strength, while the Ti-6Al-4V (Ti64) alloy has good fracture toughness, ductility, and a relatively low cost. A combination of these alloys into a single component can be used to produce advanced multimaterial parts. In this work, Ti_2_AlNb/Ti-6Al-4V bimetallic structures were fabricated from pre-alloyed powders using the Selective Laser Melting (SLM) process. The effects of high-temperature substrate preheating, post-processing by annealing, and hot isostatic pressing on defect formation, the microstructural evolution of the interface area, and the mechanical properties of the bimetallic samples were investigated. High-temperature substrate preheating during the SLM process was necessary to prevent reheat cracking of the Ti_2_AlNb part, while annealing and hot isostatic pressing post-processing improved the chemical and microstructural homogeneity of the transition zone and enhanced the tensile properties of the bimetallic structure.

## 1. Introduction

The development of next-generation aerospace and aviation components requires the use of new nonconventional material structures that provide an enhanced level of properties while allowing for a decrease in the weight of the part due to low material density and optimized topology. Additive manufacturing (AM) technologies provide the advantage of being able to fabricate geometrically complex components and combine several materials into a single part by creating bimetallic structures or functionally graded materials (FGM) [1,2,3,4].

In recent years, there has been an increased interest in the manufacture of bimetallic structures or FGM using Laser Powder-Bed Fusion (L-PBF), also known as Selective Laser Melting (SLM). One severe limitation of the process is that the existing commercial equipment is designed to use one powder material at a time, and the powder has to be replaced manually by stopping the SLM process. There have been several research papers studying the L-PBF process of bimetallic or multimaterial structures. For example, Ti/Ti-6Al-4V [5], Ti6Al4V/NiTi [6], 316L/Cu [7], and 316L/CuSn10 [8] graded structures were produced using the SLM process by alternatively using the powder of different alloys to fabricate separate areas.

Titanium Ti_2_AlNb-based intermetallic alloys exhibit high specific strength, creep resistance, serving temperatures of up to 650–750 °C, and can potentially replace nickel-based alloys or steels, which have significantly higher density [9,10,11]. However, a major problem of titanium intermetallic alloys consists in low plasticity at room temperature and poor fracture toughness [12]. The Ti-6Al-4V alloy is a widely used titanium material that has good workability and fracture toughness [13,14]. It is also considerably cheaper compared to the intermetallic Ti_2_AlNb alloy. Moreover, Ti-6Al-4V has a comparatively lower density than Ti_2_AlNb-based intermetallic alloys and can reduce the weight of aerospace parts. However, the Ti-6Al-4V alloy has limited service temperatures (below 600 °C), and its strength significantly drops at elevated temperatures.

Bimetallic or composite components consisting of two or more materials can be utilized in aerospace engine parts and offer the advantages of these materials, depending on the working conditions of the specific part [15,16]. The intermetallic Ti_2_AlNb alloy can be used for a component area that has to withstand high temperatures of 600–750 °C, while the Ti-6Al-4V alloy can be utilized to serve at lower temperatures, providing lower weight and higher fracture toughness. Thus, developing a method of producing a part that consists of these two alloys can promote the development of a new generation of aerospace engines by utilizing the full potential of these materials.

In recent years, there have been several attempts to fabricate Ti_2_AlNb/Ti-6Al-4V bimetallic joints using various techniques. Vacuum diffusion bonding using a Ti interlayer allowed for the achievement of sufficient joint strength [17]; however, this method imposes considerable limitations in terms of component geometrical complexity. The laser metal deposition AM process using TA15 and Ti_2_AlNb alloy powders was used to prepare bimetallic samples [18]. While this method has great potential in the fabrication of bimetallic titanium alloy/titanium aluminide structures, it offers substantially less possibilities in terms of complex geometrical features and worse accuracy compared to powder-bed fusion AM techniques. At the same time, the brittleness of Ti_2_AlNb alloys promotes severe cracking during laser/electron beam welding [19,20] due to high residual stresses and requires the use of high-temperature preheating. As shown in previous studies [21], the SLM process can be used to produce fully dense, crack-free Ti_2_AlNb samples when substrate preheating temperatures of 700 °C and above are applied. The feasibility of fabricating Ti_2_AlNb/Ti-6Al-4V bimetallic structures using the SLM process has not been explored so far. The issue of obtaining defect-free Ti_2_AlNb/Ti-6Al-4V bimetallic structures with a strong bond and decent mechanical properties remains unresolved.

In this study, Ti_2_AlNb/Ti-6Al-4V bimetallic structures were fabricated from pre-alloyed powders using the SLM process. The effects of substrate preheating, post-processing by annealing, and hot isostatic pressing on the microstructure of the interface area, the microhardness distribution, and the mechanical properties of the bimetallic samples were investigated.

## 2. Materials and Methods

**Figure 1:**
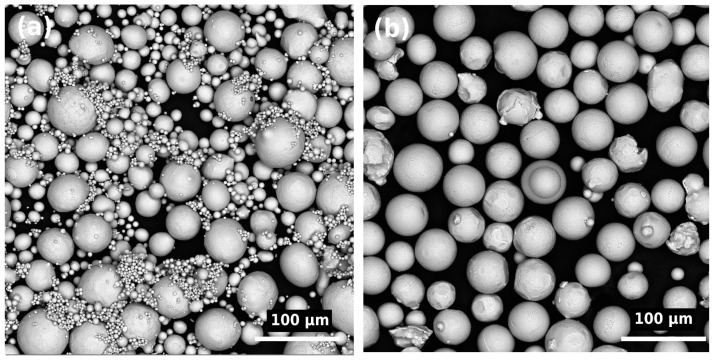
Scanning electron microscope (SEM) images of (**a**) Ti2AlNb and (**b**) Ti64 powders.

Two spherical (Figure 1) pre-alloyed powders of the Ti-6Al-4V (Ti64, Grade 5) and Ti_2_AlNb (Ti-22Al-23Nb-0.8Mo-0.3Si-0.4C-0.1B-0.2Y (at. %) alloys were used as the feedstock material in the SLM process to manufacture the samples. The Ti-6Al-4V alloy powder (Normin LLC, Borovichi, Russia) was obtained by plasma atomization with the following particle size distribution: d_10_ = 15 µm, d_50_ = 47 µm, d_90_ = 62 µm. The Ti_2_AlNb powder (AMC Powders, Beijing, China) was produced using the electron induction gas atomization process with the following particle size distribution: d_10_ = 15 µm, d_50_ = 33 µm, d_90_ = 59 µm.

AconityMIDI (Aconity3D GmbH, Herzogenrath, Germany) SLM system was used to fabricate the samples from the feedstock powders. The system is equipped with a high-temperature inductive substrate preheating, which was used to preheat the Ti-6Al-4V substrate to a defined temperature prior to starting the SLM process. The SLM process was carried out in an argon atmosphere, with the oxygen content in the process chamber kept below 50 ppm. After finishing the build process, the platform and fabricated samples were cooled to room temperature at a rate of approximately 15 °C/min.

**Figure 2:**
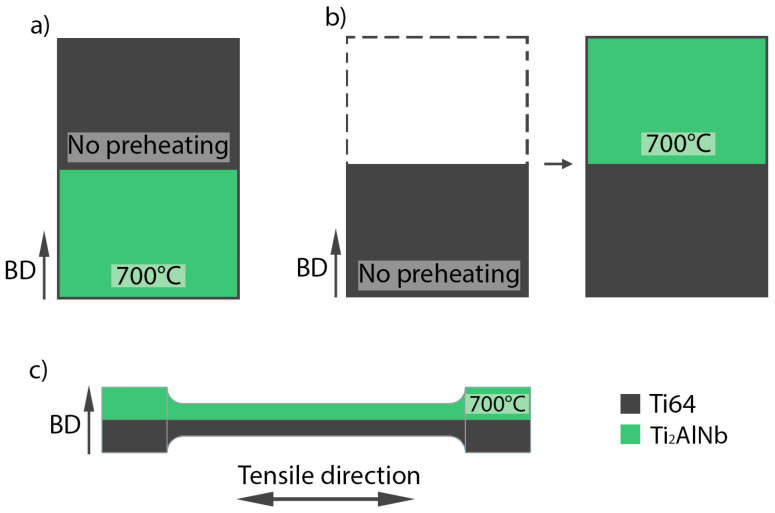
Schematic representation of SLM process options for manufacturing bimetallic sample structures: (**a**) Type A, (**b**) Type B, (**c**) tensile specimens (Type B).

Cylindrical samples with the size of ⌀10 × 15 mm were manufactured for further investigation of the microstructure, chemical composition, and microhardness. The first part of the samples was built to a specific height that corresponded to half the height of one of the feedstock powders. Then, the build process was paused, and the powder in the SLM machine was replaced with the second feedstock powder. After that, the rest of the samples was fabricated using the second powder. Two SLM process parameters in terms of platform preheating and material order variations were used to produce the samples (Figure 2):

The substrate was preheated to 700 °C, and then the Ti2AlNb alloy powder was used to fabricate the first part of the samples. Then, the platform was cooled down, the powder was replaced with the Ti-6Al-4V, which was used to fabricate the rest of the samples without applying platform preheating, labeled as Type A (Figure 2a).

The first half of the samples was fabricated from the Ti-6Al-4V powder without platform preheating. Then, the powder was replaced with the Ti2AlNb powder, the platform was preheated to 700 °C, and the rest of the samples was manufactured from the second powder, labeled as Type B (Figure 2b).

**Figure 3:**
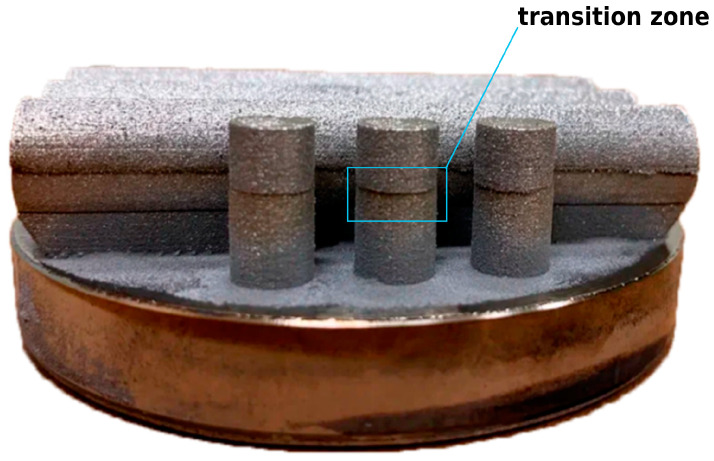
Photograph of the produced bimetallic samples showing the investigated transition zone between two alloys.

Horizontal cylindrical specimens with the size of ⌀12 × 60 mm were produced for the tensile tests according to Type B: the first half of the specimens fabricated from the Ti-6Al-4V powder and the second half using the Ti2AlNb alloy powder, with 700 °C platform preheating (Figure 2c). A photograph of the specimens is shown in Figure 3. The specimens were then machined to achieve a 25 mm gauge and a 6 mm diameter. Three samples were made for each type.

Based on previous research [5,22], the following SLM process parameters that allowed for the acquisition of fully dense samples (relative densities above 99.5%) were used:

For the Ti-6Al-4V alloy: 275 W laser power, 805 mm/s scanning speed, 120 µm hatch distance, 50 µm layer thickness, no platform preheating.

For the Ti2AlNb alloy: 140 W laser power, 850 mm/s scanning speed, 120 µm hatch distance, 30 µm layer thickness, 700 °C platform preheating.

A standard metallographic sample preparation technique was used to prepare the samples for microstructural investigation, which included cutting the sample along the build direction, grinding, and polishing the cut sample.

The microstructure of the samples was studied using cut and polished sections, with the use of a Mira 3 (TESCAN, Brno, Czech Republic) scanning electron microscope (SEM) using the back-scattered electron (BSE) mode. An energy dispersive X-ray (EDX) spectroscopy module was used to evaluate the chemical composition of the samples.

Tensile tests were carried out using the Zwick/Roell Z100 (Zwick GmbH, Ulm, Germany) testing machine. The tensile direction was perpendicular to the build direction, while the material change line was parallel to the tensile direction.

The manufactured samples were additionally heat-treated in a vacuum furnace by annealing at 1050 °C for 1.5 h, followed by furnace cooling.

Additionally, the samples were subjected to hot isostatic pressing (HIP) using the following parameters: 1160 °C temperature, 3 h holding time, and 160 MPa pressure.

## 3. Results and Discussion

**Figure 4:**
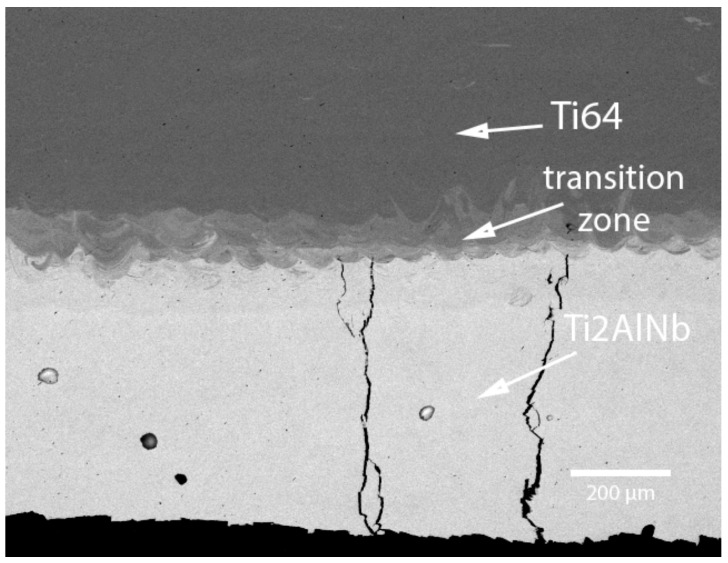
BSE image of the bimetallic sample produced without platform preheating (Type A).

Figure 4 shows the BSE-SEM image of the Ti2AlNb/Ti64 bimetallic structure, where the Ti64 part was built on top of the Ti2AlNb part without applying platform preheating (Figure 2a). It can be seen that without preheating, severe cracking occurs in the Ti2AlNb part when the Ti64 material is deposited on top of it. The cracks are parallel to the build direction and have a length of up to several mm. The formation of cracks in the Ti2AlNb area occurs due to the high brittleness of the alloy coupled with high residual stresses induced by periodic rapid heating and cooling thermal cycles during the SLM process. A distinct transition zone can be seen in the BSE image due to the chemical difference between the Ti2AlNb-based alloy and the Ti64 alloy. The transition zone has a width of approximately 100–150 µm, which corresponds to the thickness of 2–3 layers. In the case of the bimetallic sample produced with platform preheating and by building the Ti_2_AlNb alloy on top of the Ti64 alloy (Figure 2b), no cracking of the material occurred.

**Figure 5:**
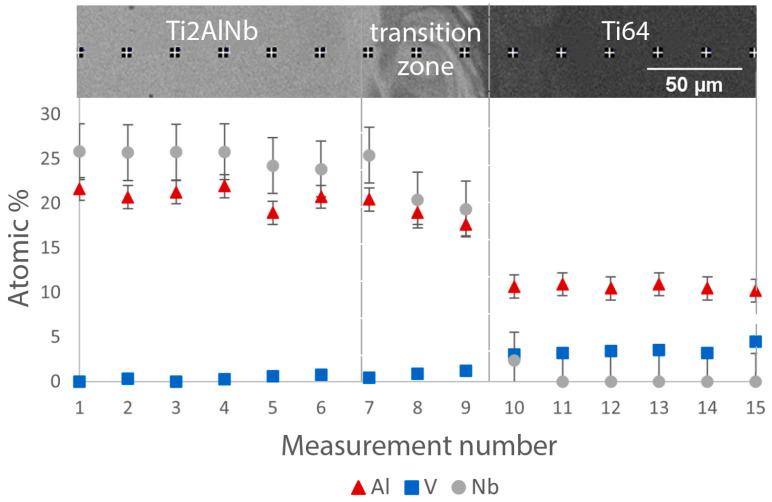
Variation of Al, V, and Nb content along the transition zone for the bimetallic sample produced with platform preheating (Type B).

Figure 5 shows the variation of the Al, V, and Nb content along the transition zone for the bimetallic sample produced with platform preheating. A steep decrease in Nb content and an increase in V content in the transition zone can be seen for the transition from the Ti_2_AlNb to the Ti64 alloy.

**Figure 6:**
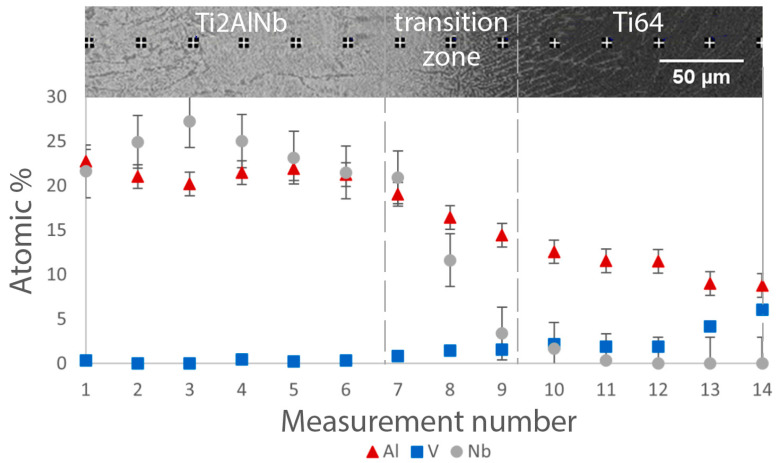
Variation of Al, V, and Nb content along the transition zone for the bimetallic sample produced with platform preheating after annealing (Type B).

Figure 6 shows the variation of the Al, V, and Nb content along the transition zone in the annealed sample. Compared to the as-fabricated condition, the change in the main alloying element content is smoother. The Nb content uniformly decreases in the transition zone from the Ti_2_AlNb to the Ti64 alloy, while the V content smoothly increases. Thus, annealing resulted in a more uniform change in the element content in the transition zone due to diffusion processes. Similar elemental variation was obtained for the HIPed sample.

**Figure 7:**
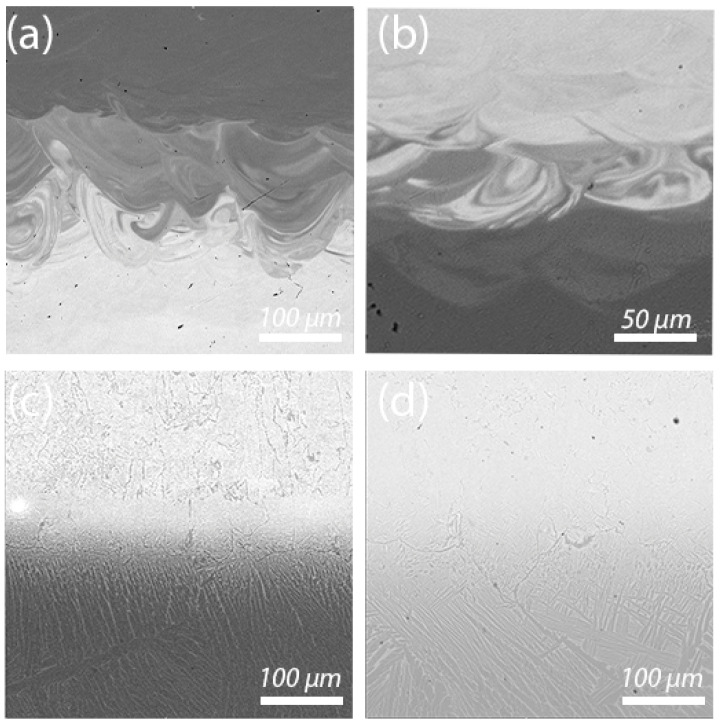
BSE-SEM images showing microstructures of the bimetallic samples: (**a**) fabricated without platform preheating (Type A), (**b**) fabricated with platform preheating (Type B), (**c**) Type B after annealing, (**d**) Type B after HIP.

The microstructures of the bimetallic samples in as-fabricated, annealing, and HIPed conditions are shown in Figure 7. In the as-fabricated condition, the Ti64 zone features a fine α′-martensite microstructure induced by high cooling rates during the SLM process [23,24], and the Ti_2_AlNb zone has a single-phase Ti_2_AlNb microstructure (Figure 7a). The thickness of the transition zone is approximately 100–150 µm. The transition zone features chemical inhomogeneity in form and an uneven Nb distribution, as can be seen in the BSE-SEM image. Lighter areas correspond to higher Nb content, while darker areas correspond to lower Nb content. After annealing, the α′-phase decomposes into α- and β-phases, forming a lamellar structure, and the transition zone composition becomes more uniform (Figure 7c). The diffusion of Nb increased as a result of annealing, which led to the formation of the lamellar α + β structure since Nb acts as a β-stabilizing element. The Ti_2_AlNb area consists of a dual-phase β/B2 + Ti_2_AlNb microstructure after annealing. The HIP of the bimetallic sample resulted in a uniform transition from the α + β microstructure for the Ti64 to the β/B2 + Ti_2_AlNb microstructure for the Ti_2_AlNb alloy area (Figure 7d). After HIP, the distinction between the two alloys in the transition area became visible due to the greater distance in the Nb diffusion since the HIP was carried out at a higher temperature than annealing.

**Figure 8:**
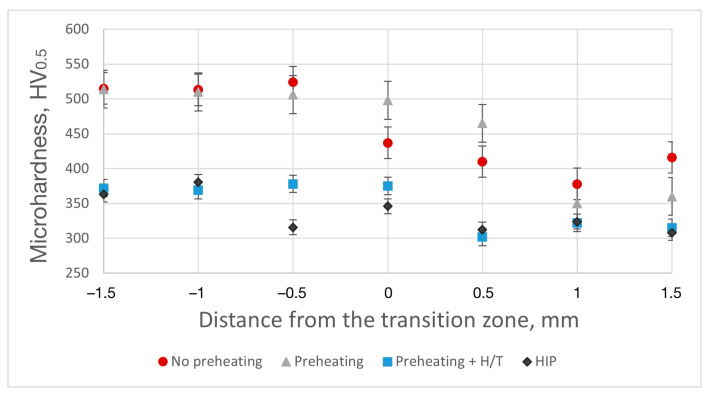
Variation of microhardness along the transition zone of as-fabricated, annealed, and HIPed bimetallic structures.

As shown in Figure 8, microhardness variation along the transition zone of the Ti64/Ti_2_AlNb alloy bimetallic structure is significant, depending on the SLM processing conditions as well as post-treatment. The bimetallic structure in the as-fabricated condition shows a significant microhardness difference between the Ti64 and Ti_2_AlNb alloy zones. The samples fabricated without platform preheating features the Ti_2_AlNb zone with higher microhardness compared to the Ti64 zone. This is attributed to the intermetallic Ti_2_AlNb phase having higher microhardness compared to the α’-Ti phase. The single-phase Ti_2_AlNb zone has a microhardness of around 510 ± 20 HV, which is similar to the values obtained for the single-material SLMed Ti_2_AlNb alloy [21]. When platform preheating was applied, the microhardness of the Ti_2_AlNb zone decreased significantly as its microstructure changed to the β/B2 + Ti_2_AlNb phases, which are characterized by lower microhardness compared to the single-phase Ti_2_AlNb microstructure. The annealing and HIP of the bimetallic structure eliminated a steep change in microhardness between the two alloys and resulted in a uniform microstructure along the transition zone. The resulting uniform microhardness distribution is associated with microstructure transformation to the β/B2 + Ti_2_AlNb and α + β microstructures in the Ti_2_AlNb and Ti64 zones, respectively, as well as with elemental diffusion and a more uniform elemental distribution.

The obtained tensile properties for the Ti64/Ti_2_AlNb bimetallic structures as well as the single Ti64 and Ti_2_AlNb alloys are presented in Table 1. The produced bimetallic structures’ room-temperature tensile strength (TS) and yield strength (YS) are limited by the material with the lowest strength. At the same time, the high-temperature strength of the bimetallic structure is higher than that of the single Ti64 alloy obtained by Direct Laser Deposition by approximately 35% in the HIPed condition, but lower than that of the single Ti_2_AlNb alloy. HIP allowed for a significant enhancement of the tensile properties of the bimetallic structure as it significantly improves the strength and elongation of the Ti_2_AlNb alloy due to microstructural changes and the elimination of residual porosity.

## 4. Conclusions

In this research, Ti_2_AlNb/Ti-6Al-4V bimetallic structures were fabricated using the SLM process. High-temperature platform preheating during the SLM process was necessary to eliminate the cracking of the intermetallic alloy. The transition zone had a distinct microstructure, with an inhomogeneous elemental distribution and a thickness of 100–150 µm. The application f platform preheating as well as post-processing by annealing and hot isostatic pressing significantly affected microstructure and microhardness variation along the transition zone. Annealing and hot isostatic pressing resulted in a more uniform microstructural transition between the Ti_2_AlNb and Ti64 zones, while microhardness differences between the zones were almost eliminated. After annealing and hot isostatic pressing, the α′-phase in the TI64 alloy decomposed into α- and β-phases, thus forming a lamellar structure, while the dual-phase β/B2 + Ti_2_AlNb microstructure formed in the Ti_2_AlNb alloy zone. Diffusion of the Nb promoted by annealing and hot isostatic pressing post-processing allowed us to achieve a uniform transition between the Ti64 and Ti_2_AlNb zones.

The high-temperature strength of the bimetallic structure was higher than that of the single Ti64 alloy by approximately 35%, but lower than that of the single Ti_2_AlNb alloy. The tensile strength of the bimetallic structure after hot isostatic pressing reached 1020 MPa and 515 MPa at room temperature and 650 °C, respectively.

Future investigations in multimaterial additive manufacturing should be focused on the study of mechanical properties in different directions relative to the building direction as well as on the creation of interfacial changes with a smooth change in chemical composition and an increased transition zone.

## Figures and Tables

**Table 1:** Tensile properties of Ti64, Ti_2_AlNb alloys and Ti_2_AlNb/Ti-6Al-4V bimetallic structures.

Material	Testing Temperature, °C	YS, MPa	TS, MPa	Elongation at Break, %
Ti64 SLM [25]	20	1200	1280	2.4
Ti64 SLM + annealing [25]	20	821	998	10
Ti64 SLM [25]	350	892	979	6.3
Ti64 SLM + annealing [25]	350	615	745	13.1
Ti_2_AlNb SLM + annealing [22,26]	20	-	630	-
Ti_2_AlNb SLM + HIP [22,26]	20	-	1090	1
Ti_2_AlNb SLM + annealing [22,26]	650	-	647	-
Ti_2_AlNb SLM + HIP [22,26]	650	797	875	4
Ti_2_AlNb/Ti64 SLM + annealing	20	-	689	0.6
Ti_2_AlNb/Ti64 SLM + HIP	20	920 ± 20	1020 ± 20	1.9 ± 0.1
Ti_2_AlNb/Ti64 SLM + HIP	650	470 ± 10	515 ± 15	3 ± 0.3
Ti_2_AlNb/TC11 (electron beam welding) [27]	20	1030	1100	13
Ti64 (Direct Laser Deposition) [28]	700	-	~380	~7

## Data Availability

The data presented in this study are available on request from the corresponding author.
